# From Pixels to Prediction: Reviewing the Role of Artificial Intelligence in Body Composition Analysis

**DOI:** 10.1002/jcsm.70218

**Published:** 2026-05-18

**Authors:** Marta Zerunian, Benedetta Masci, Stefano Nardacci, Federico Perconti, Lapo Nardoni, Vincenzo Solimene, Michela Polici, Francesco Pucciarelli, Tiziano Polidori, Domenico De Santis, Andrea Laghi, Damiano Caruso

**Affiliations:** ^1^ Department of Surgical and Medical Sciences and Translational Medicine Sapienza University of Rome, Sant'Andrea University Hospital Rome Italy; ^2^ PhD in training, PhD School in Translational Medicine and Oncology, Department ofMedical and Surgical Sciences and Translational Medicine, Faculty of Medicineand Psychology Sapienza University of Rome Rome Italy; ^3^ Department of Biomedical Sciences Humanitas University Milan Italy; ^4^ Department of Diagnostic Imaging IRCCS Humanitas Research Hospital Milan Italy

**Keywords:** artificial intelligence, body composition, clinical application, risk prediction

## Abstract

Evaluation of body composition (BC) is a set of biomarkers, including fat, muscle and bone, that allows the quantification of an individual's composition at different levels of complexity (whole body, tissues, macroscopic and microscopic). Recently, BC has gained medical interest due to its impact on health outcomes in both oncologic and non‐oncologic conditions. BC parameters are also valuable for opportunistic screening, offering benefits to patients in prevention and health management. Among non‐invasive methods to assess BC, cross‐sectional imaging, for example, tomography (CT) and magnetic resonance (MR), can provide quantitative data from exams conducted for other reasons that can significantly aid large‐scale health prevention. Recent studies highlight BC's potential in personalized medicine, but challenges like lengthy and poorly repeatable manual segmentation and complex analysis have limited its routine clinical use. Artificial intelligence (AI) can address these issues by simplifying and automating the process, from segmentation to prediction (e.g., automatic muscle segmentation for sarcopenia assessment and AI assessment of BC as opportunistic evaluation during CT scan for other clinical reasons). This review aims to summarize recent advancements in BC and AI, showcasing their synergy in enhancing the management of health conditions, from diagnosis to personalized treatments, while discussing current limitations and future challenges.

## 1 Introduction

BC has increased in importance in order to analyse several pathological conditions, both non‐oncological and oncological pathologies [1]. Knowing how much a person weighs is far less informative than understanding what that weight consists of and where it is distributed, involving different physiopathological mechanisms that interact with complex pathways, including hormonal and metabolic regulation [2]. In fact, the possibility to measure the distribution of different types of adipose tissue, lean mass and bone tissue has been proven to play a pivotal role in the risk stratification, prognostic role, and therapeutic monitoring in chronic complex pathologies such as metabolic syndrome, obesity, diabetes, and cardiac disease [3]. Furthermore, BC is increasingly taking on an integrated role in the management of oncological diseases, providing important prognostic information and overall patient assessment, including in relation to treatment choices and patient outcomes [4]. Various methods are used to assess BC, including anthropometry, densitometry, and bioelectrical impedance analysis [5]. However, imaging techniques like computed tomography (CT) and magnetic resonance imaging (MRI) are considered higher‐performance methods because of their capacity to analyse tissue and organs [6]. In particular, automatic BC analysis (BCA), including assessment of muscle, fat and bone tissues during follow‐up, enables the quantitative monitoring of its modifications with the disease course [7]. For instance, BCA is useful for sarcopenia assessment by evaluating skeletal muscle mass from diagnostic imaging, either obtained for BCA purposes or opportunistically extracted in clinical imaging performed for other clinical questions. This approach enables reliable detection of sarcopenia, defined as progressive loss of skeletal muscle and functional decline [8], associated with increased risks of falls and bone fractures, disability, and mortality [9], especially in older adults and oncologic patients [10].

Furthermore, it has been shown that socioeconomic disadvantage is associated with BC measurements over or below highly specific established thresholds per parameter [11] predicting mortality from cardiovascular and metabolic diseases.

Technological implementations are also involving BC assessment at different levels of analysis. Different studies suggest that CT‐derived BCA would improve BCA accuracy and repeatability by providing data of multiple tissue compartments derived from high‐resolution volumetric CT imaging; this enables a precise quantification of different body components, maximizing the information contained in a single diagnostic examination and reducing the need for additional (direct or indirect) BC methods [12].

However, even in this promising scenario, the clinical application of BC is still limited due to some drawbacks, among which are the time‐consuming nature of the segmentation, the reduced inter‐reader agreement and the lack of measurement standardization [13]. To overcome these limitations, artificial intelligence (AI) represents a powerful tool to narrow the gap and disseminate clinical use. In fact, AI methods offer automated and highly reproducible segmentation tools that can improve BCA based on training with large annotated datasets for BC assessment, overcoming the need for manual or semi‐automatic segmentation and enabling a wider spread of clinical implementation [14]. Furthermore, beyond automatic segmentation, AI models are reaching more complex aims, such as integrating imaging features, clinical and molecular biomarkers, supporting more translational personalized risk assessment and treatment planning [15].

This review aims to outline BC principles, imaging techniques and clinical utility. A special focus will also be provided on AI innovation and on its synergy with BCA, pointing out real‐life problems and possible solutions, current drawbacks, and future directions for actual AI‐based BCA applications.

## 2 Body Composition Parameters in Brief

BC is the ultimate result of the human body's elements quantification. For decades, scientific research has focused on BC assessment, proposing a five‐level mode: (i) atomic; (ii) molecular; (iii) cellular; (iv) tissue, which incorporates skeletal muscle mass (SMM), adipose and lean tissue; and (v) whole body, combining the mass of skeletal segments [16]. The ideal BC model for a true evaluation is surely the three‐compartment model, which further divides body mass into fat, lean and bone mass [17].

### 2.1 Fat

Fat represents a complex and heterogeneous compartment, comprising different subtypes that differ in terms of location and integration into the patient's metabolism. Since then, most studies have focused on the assessment of body fat [18], which can be distinguished into subcutaneous adipose tissue (SAT) and visceral adipose tissue (VAT), based on anatomical location [19]. In particular, the VAT can be further divided into specific visceral depots (e.g., mesenteric fat and retroperitoneal fat), with a peculiar impact on different metabolic pathways [20].

The function of the SAT is to store excess fat, which, once saturated, leads to an accumulation of VAT [21]. Furthermore, increased VAT deposition leads to an accumulation of ectopic fat in tissues such as the liver, heart, muscles and skeleton [21]. VAT accumulation (and consequently ectopic fat) represents a serious risk to human health as it contributes to the development of several pathological conditions [22]. For instance, increased pericardial fat volume has a negative impact on some cardiovascular diseases, including coronary artery disease or atrial fibrillation, as emerged from an interesting meta‐analysis performed on 83 studies [23]. Furthermore, an excess of intrahepatic adipose storage negatively influences insulin resistance and systemic inflammation and determines increased fibrotic changes of the liver parenchyma with increased risk of developing HCC, particularly in hepatic steatosis part of metabolic dysfunction‐associated steatotic liver disease [24]. As well as increased perirenal fat accumulation affects glomerular filtration rate and the onset of chronic kidney disease. The impact of fat in this case seems to be double: a ‘mechanical’ effect on neighbouring vascular and lymphatic structures, and from a systemic point of view, through the production of specific cytokines and metabolites negatively correlated with chronic kidney disease, overcoming simpler fat assessment evaluation such as VAT and SAT [25]. Among ectopic fat, also intermuscular fat increases muscle metabolic dysfunction and sarcopenia development [26].

A rapid body fat assessment can be carried out using the body mass index (BMI) [18], defined as weight normalized by the square of the patient's height (kg/m^2^), categorizing a person into six groups [27]. Although widely used in clinical practice, BMI assessment cannot differentiate lean mass from fat mass, and more tailored tools that are easy to use in clinical practice are warmly encouraged [28].

### 2.2 SMM

SMM is another core parameter of BCA. It accounts for approximately 40% of total body weight and is influenced by nutritional status, physical activity, endocrine milieu and diseases [29]. Muscle characterization with several different parameters allows different pathophysiological aspects to be identified, with significant clinical implications. Besides muscle quantification, muscle quality assessment, including fat infiltration (myosteatosis) and contractile status, has a high impact on the SMM characterization, including its function and strength [30].

As age increases, SMM undergoes complex changes also related to type‐II fibre atrophy, low‐grade inflammation and motor‐unit loss with denervation–reinnervation, which contribute together to reduce strength, power and fatigue resistance [29]. These changes led to different forms of alterations, including myopenia, characterized by low muscle mass [8], and the more clinically relevant sarcopenia, defined as low muscle strength with confirmation by low muscle mass/reduced muscle attenuation and severity by poor physical performance [31]. Two types of sarcopenia are recognized in clinical practice: primary sarcopenia, age‐related loss of muscle mass and secondary sarcopenia, related to loss of muscle mass due to chronic diseases, cancer or heart failure [32].

SMM evaluation is also deeply involved in the diagnosis of cachexia [33]. It is defined as ‘a complex metabolic syndrome associated with underlying illness and characterized by loss of muscle with or without loss of fat mass’ [34] and represents a multifactorial metabolic syndrome, driven by disease (e.g., cancer) and systemic inflammation that cannot be fully reversed by conventional nutritional support. Clinically, cachexia is correlated to reduced treatment tolerance and survival, underlining the need to precisely assess muscle loss in chronic disease.

### 2.3 Bone

In the three‐compartment model, in addition to the assessment of fat mass and lean mass, bone mineral content (BMC) is also estimated, representing the skeleton's mineral fraction, usually named as BMC. It is also important to point out that BMC is a measure expressed in grams, which distinguishes it from bone mineral density (BMD) expressed as a concentration measure (g/cm^2^), which reflects mineral per unit area and is used to estimate skeletal strength. The two measurements are related but not interchangeable. In this context, assessing the bone compartment is crucial to recognize variation that can become pathological. In fact, the reduction in the bone compartment signifies loss of osseous mass and consequent mineral store, with changes in the bone microarchitecture and remodelling balance, strictly linked with elevated fracture risk, poor quality of life and increased risk of mortality [35]. The bone compartment has several interactions with the other compartments inasmuch as skeletal muscle supports bone through mechanical loading and biochemical signalling while adipose tissue influences bone via adipokines and adipose content of bone marrow.

### 2.4 Fat–Muscle–Bone Axis

All three major compartments, globally enlisted as adipose tissue, skeletal muscle and bone, intersect with each other through endocrine (adipokines/myokines/osteokines), inflammatory and biomechanical pathways. In this scenario, also pathologic alterations are intrinsically tightly bound, with the coexistence of osteosarcopenia, and in the case of the presence of excess adiposity, osteosarcopenic obesity. The coexistence and mutual interactions of the different impaired compartments lead to greater frailty, falls and fractures than any single deficit per se, supporting concurrent assessment and integrated interventions [35].

## 3 Imaging Techniques at a Glance

The gold standard in BC evaluation is autopsy examination, but because direct in vivo assessment is not possible, indirect methods must be used. In this scenario, due to imaging‐derived body‐composition metrics increasingly informing risk stratification and treatment decisions, not only radiologists but also clinicians should grasp the basic principles of CT, MRI, and DXA applicable to BC in order to deepen the knowledge about each method's potential and limitations.

### 3.1 DEXA

The most used method for assessing BC is dual‐energy X‐ray absorptiometry (DEXA), which allows the evaluation of the three compartments: fat, lean soft tissue (LST) and bone [36]. DEXA is based on the attenuation of X‐rays emitted at two different energy levels by the tissue, whose attenuation coefficient is used to differentiate between LST (including lean mass) and fat and bone mass [37]. In clinical practice, DEXA is used to assess BMD, but it can also be extended to assess lean body mass and body fat of a single region or the whole body [18]. However, DXA showed an important limitation in the assessment of LST related to hydration. In fact, by design, LST includes tissue water and assumes a constant hydration (~73%). When hydration deviates, such as in cases of oedema/ascites, inflammatory states, heart/renal/liver failure or recent endurance exercise, the extra water is included in the calculation of LST, with consequent overestimation of lean mass (including appendicular LST), reducing the accuracy of lean mass assessment and reproducibility [37]. Therefore, the DXA method for BC should be interpreted with caution in the case of fluid‐overloaded patients, who might request repeated measurements in euvolemia condition, and, when needed, complemented by CT/MRI‐based BC assessments, less sensitive to hydration bias [38].

### 3.2 CT

**FIGURE 1:**
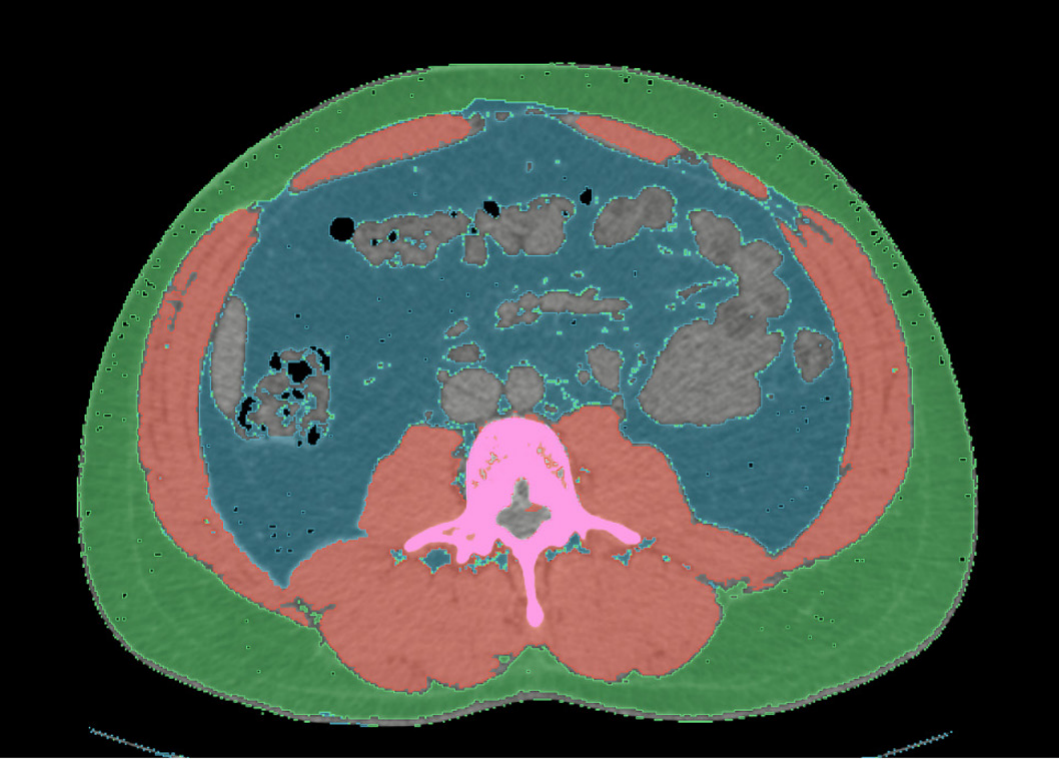
Segmentation of a CT axial image at the L3 lumbar vertebra level using Hounsfield units (HU): Subcutaneous adipose tissue is identified with a threshold between −190 and −30 HU (green), visceral adipose tissue between −150 and −50 HU (blue) and skeletal muscle tissue between −29 and 150 HU (red). Higher HU values (> 400) can detect bone (pink). The segmentation is performed with an open‐source software (http://www.slicer.org, version 5.6.2, revision 32448, built 2024‐04‐05).

CT, particularly of the abdominal sections, is widely used in clinical practice for several indications (i.e., abdominal emergencies, pre‐surgical planning, oncologic at baseline and follow‐up to assess primary and secondary tumoral localizations, inflammatory bowel disease assessment) and provides thousands of volumetric images with a multitude of information beyond radiographic data. Therefore, from CT images acquired for other reasons, BC data such as quantification of VAT, SAT, BMD, muscle mass and aortic calcifications can be opportunistically and retrospectively obtained [39]. A CT scan uses an X‐ray beam that passes through body tissue and is collected by a detector. This data is then used to create an image with each pixel showing densitometric information in Hounsfield unit (HU) [40]. Each tissue presents HU according to the attenuation of the X‐ray beam (Figure 1). Using these HU thresholds, tissue can be separated and BC derived through the various segmentation methods available [41]. A single axial landmark at the level of the L3 lumbar vertebra has been identified as the main one, because at this level, the abdominal musculature, psoas, paravertebral muscles, SAT and VAT tissue can be assessed [42]. Furthermore, CT can provide information about intramuscular fat accumulation and, to a lesser extent, in the liver, with a limited sensitivity in low‐grade steatosis (< 5%) [18].

The main disadvantage of the CT scan is surely the extensive use of ionizing radiation and the high costs of the examination [38], as well as the training and the time required to perform the image segmentation process, which would benefit from an AI‐driven method to speed up without affecting accuracy [18].

### 3.3 MRI

MRI is considered a valuable method to assess BC. As well as CT technique morphological MRI sequences imply the acquisition of multiple slices of a body section (e.g., abdomen or whole body MRI) that need precise segmentations to separate different BC compartments to extract data. Similarly to CT, there is a need to automate the segmentation process, which becomes even more complex in resonance imaging as there are no fixed signal intensity thresholds to apply, using highly accurate tools such as those that can be developed using AI. Furthermore, quantifying adipose tissue and lean mass, or organ adipose infiltration can be done with quantitative MRI, by using fat–water imaging techniques, like Dixon sequences [18], allowing the quantification of muscle fat or VAT infiltration, as in steatosis [43]. However, there are factors that can affect the fat fraction signal generated by these sequences and thus alter fat quantification. Therefore, sequences with reconstruction algorithms have been proposed to overcome these biases, such as proton density fat‐fraction (PDFF) [44]. Examples of these sequences are shown in Figure S1a,b.

Unfortunately, this technique is not widely used given the high costs and the inaccessibility of the technique to those with non‐compatible metal devices [42].

All this evidence is summarized in Table S1.

## 4 Clinical Impact of Measuring Body Composition

BCA has significant clinical impact aiding overall health assessment and prediction of several adverse events [45, 46]. The rationale relies on the identification of indexes and conditions like osteoporosis, obesity and sarcopenia that help in monitoring treatment efficacy and personalizing treatments for chronic diseases [47].

Sarcopenia and adipose tissue distribution are considered key risk factors for overall survival, postoperative complications and treatment‐related toxicity in cancer patients [48]. Bundred et al. [49] reviewed 42 studies showing that sarcopenia increases the peri‐operative mortality (OR: 2.40, 95% CI 1.19–4.85, *p* < 0.01) and reduces survival. Moreover, Troschel et al. [41] linked low muscle mass in lung cancer patients to worse prognosis and higher perioperative complication risk.

Muscle mass evaluation in oncology has demonstrated implications for treatment‐related toxicity due to different factors such as the low volume of distribution of drugs and the consequent therapy overdose or the hepatic cytochrome impairment related to the pro‐inflammatory states that impact the drug metabolism [50]; as suggested by Tan et al. [51], sarcopenia was considered a predictor of dose‐limiting toxicity in oesophagogastric cancer patients. Moreover, Huillard et al. [10] reported early dose‐limiting toxicities in sarcopenic renal cell cancer patients. In terms of muscular fatty infiltration or myosteatosis, a meta‐analysis by Aleixo et al. [52] found muscle fatty infiltration linked to shorter survival and a 75% higher mortality risk in gastrointestinal, pancreatic and hepatic cancers.

Regarding VAT, its increased representation has often been associated with reduced survival in breast cancer patients [53] and in patients with metastatic chemotherapy‐resistant prostate cancer [54]. BC evaluation also benefits non‐oncological conditions by assessing therapy impact and identifying risk factors.

Muscle mass assessment is crucial in diabetes and metabolic syndrome due to its negative prognostic link with osteoporosis [9, 46, 55]. Moreover, assessing fatty infiltration in pelvic, thigh and leg muscles helps evaluate functional ability and insulin regulation in osteoarthritis and neuromuscular disorders [56].

Regarding cardiologic patients, increased abdominal fat strongly correlates with higher cardiometabolic risk [57, 58]. Moreover, increased epicardial adipose tissue was independently associated with augmented arterial stiffness [59] and left ventricle remodelling [60]. Todd et al. [61] linked higher intrathoracic fat volume to myocardial contractile dysfunction in coronary artery disease patients.

BCA is essential for evaluating contrast doses in CT, significantly affecting tissue segmentation [62]. It has been shown that muscle and fat attenuation variations influence parameters like SMI and steatotic muscle area, which, in turn, are also dependent on the contrast injection phase [63, 64]. Contrast media, rather than radiation dose, can have an impact on these parameters. Additionally, thicker CT slices reduce SMI and increase steatotic muscle area and adipose index, but less than contrast phases [62, 63]. Lean body weight‐based contrast dosing and spectral CT can lower dosages while maintaining image quality [65]. However, it is crucial to keep in mind that, depending on the enhancement phase, measurements of SMM and density can vary statistically significantly due to contrast enhancement alone [64]. Therefore, this aspect shows how contrast‐enhanced imaging and BCA are mutually influenced: as the precise assessment of the different compartments influences the administration of contrast medium and therefore enhancement, which in turn influences the accuracy of BCA assessment.

## 5 Current Limitations of Body Composition: Why are We Not Exploiting It?

Thus far, BC measures provide quantifiable information about tissue distribution, allowing for customized treatment of a range of illnesses [46]. Notwithstanding these developments, BCA still has significant drawbacks, such as lack of standardization, inconsistent measurements and technological difficulties [45, 66]. For instance, BC tools are sometimes expensive, scarce and require expert staff, which restricts their regular use [14, 47].

**FIGURE 2:**
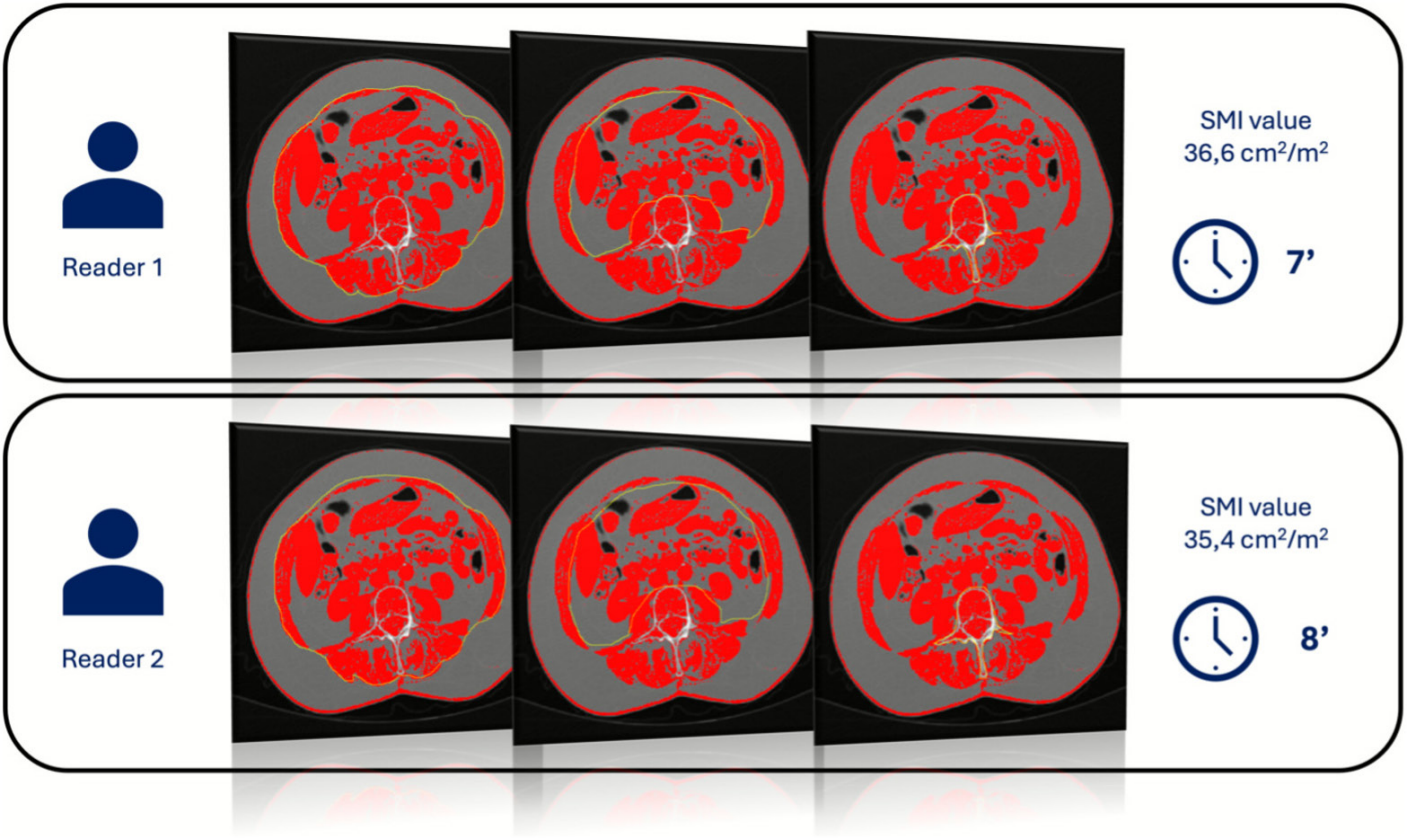
Example of a semi‐automatic segmentation method (open‐source software ImageJ, available online: https://imagej.net/ij/nih‐image). Two different readers performed the segmentation of the outer perimeter of the abdominal musculature (yellow line), the inner perimeter of the abdominal musculature (yellow line) and the vertebral body (yellow line), obtaining two different values of skeletal muscle index (SMI). The method, besides being time consuming, also showed different values of SMI that differed almost by one point with high risk of bias for further analysis.

Technological challenges include imaging issues, as described in the previous paragraph, and reliance on manual or semi‐automatic segmentation, which is time consuming [45], prone to human errors and variability and potentially overestimates parameters like muscle mass, highlighting the need for automated tools [66] (Figure 2).

BC protocols often lack standardization, complicating consistent cut‐offs and comparisons across studies. In fact, study variability largely hampers data comparison and guideline development. A meta‐analysis from Price et al. [66] observed that the vast majority of the studies included were single centred, limited by a small sample size and used varying CT acquisition protocols, leading to eventual biases in the segmentation processes.

Overcoming the current limitations of BC assessment with technological advancements such as AI could certainly improve the use of it in clinical practice, improving its potential benefits for personalized healthcare [14].

## 6 What a Clinician Should Know About AI

AI has significantly advanced in many aspects of life, including medicine. Despite its potential, concerns about AI replacing human roles in medicine persist, warranting clarification.

**FIGURE 3:**
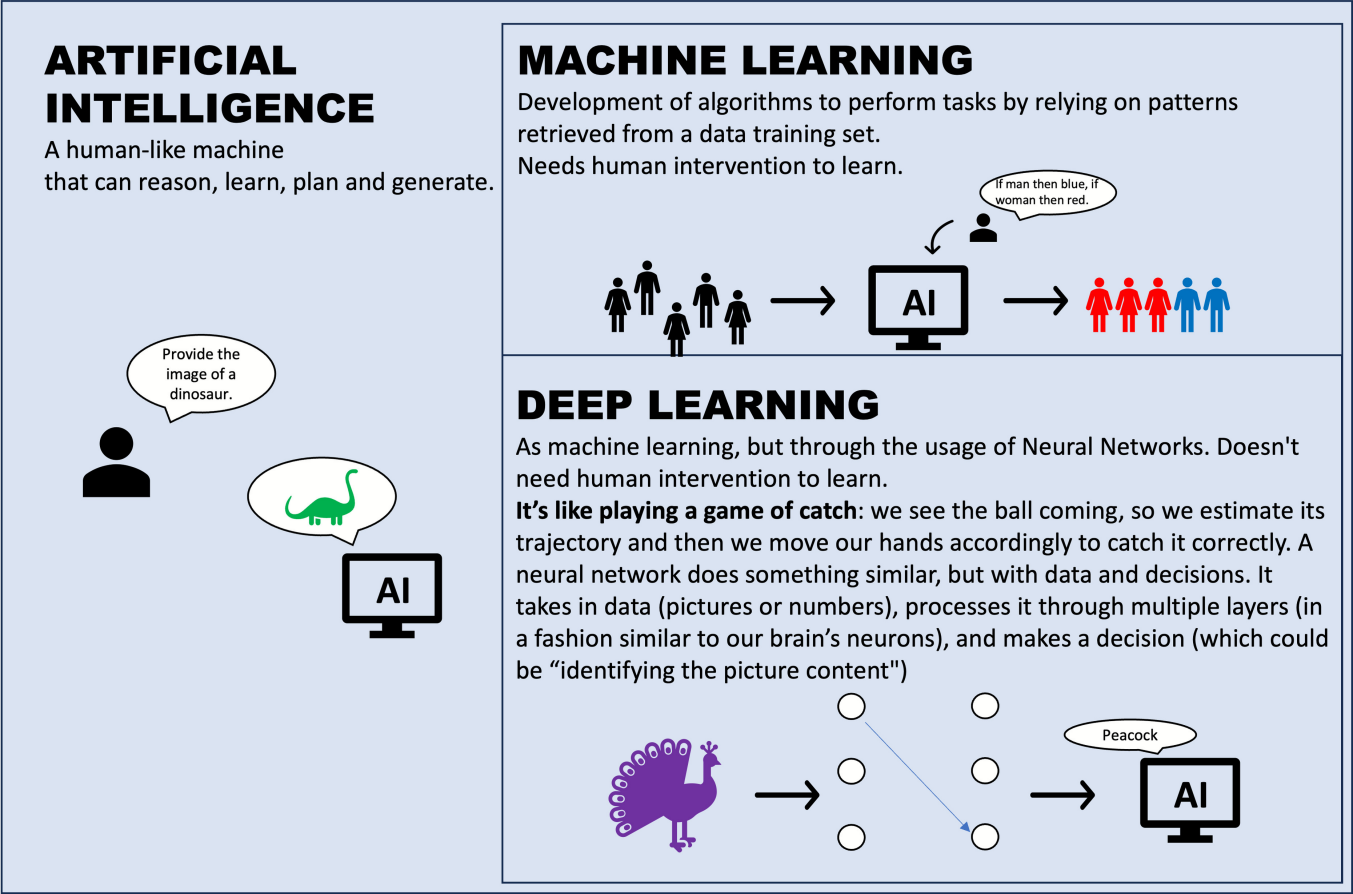
A schematic depiction of the relationships between artificial intelligence (AI), machine leaning (ML) and deep learning (DL). AI embraces deeply our routines nowadays, it is a specific algorithm that has been trained with ML to recognize a specific information from a set of data (that can contain billions of instructions), giving an output based on the combination of all the available knowledge. DL is the name given to this specific process; DL employs the so called neural networks (NN) that represent the layers through which all the nodes go. As we know that these nodes exist, we do not know the exact path of each piece of data: This is why NN are often referred as «black box».

The European Parliament defines AI as machines exhibiting human‐like reasoning and learning, with feedback‐based improvement crucial in medicine. The term AI represents a term that encompasses many subsystems with different functions, and for the purposes of this review, we will focus more on DL and ML, which are the most commonly used models in the medical field and specifically in BCA tools. Its most exploited ability is that of being able to learn and constantly improve the given answers (output) from the processed feedback (input), introducing machine learning (ML) and deep learning (DL). ML enables computers to learn without explicit programming using algorithms and statistical models [67], whereas DL, a subset of ML, processes raw data into higher outputs using neural networks [68], as shown in Figure 3 in association with a real‐life analogy helpful to easily understand the basic principle behind this [69]. In simpler terms, the differentiation between ML and DL is rooted in the structure of the models and the intricacy of the data they can process: ML models generally depend on feature engineering and might not be adequate at managing unstructured data; on the other hand, DL models possess the capability to autonomously learn hierarchical data representations, rendering them suited for processing unstructured data such as images and text [70]. For this reason, DL algorithms have shown remarkable efficiency in tasks like image analysis, including object detection and recognition. Nonetheless, DL algorithms are utilized in a black box approach, offering little to no clarity about the elements that shape their evaluations [71].

Nowadays, AI is already widespread in radiology, even if we do not fully realize it: many algorithms are used to optimize patients' worklists, to automatically highlight pathologic findings [72] or quality improvement of CT/MRI images [73]. In this context, it is important to remember that the radiological practice consists of multiple elements beyond the mere interpretation of images, ranging from the assessment of prescriptive appropriateness to the clinical anamnestic correlation with the prescribing clinician [74]; therefore, it is clear that AI in the field of image analysis can only serve as an aid to radiologists in implementing the workflow and will never be an enemy competing to replace his role.

**FIGURE 4:**
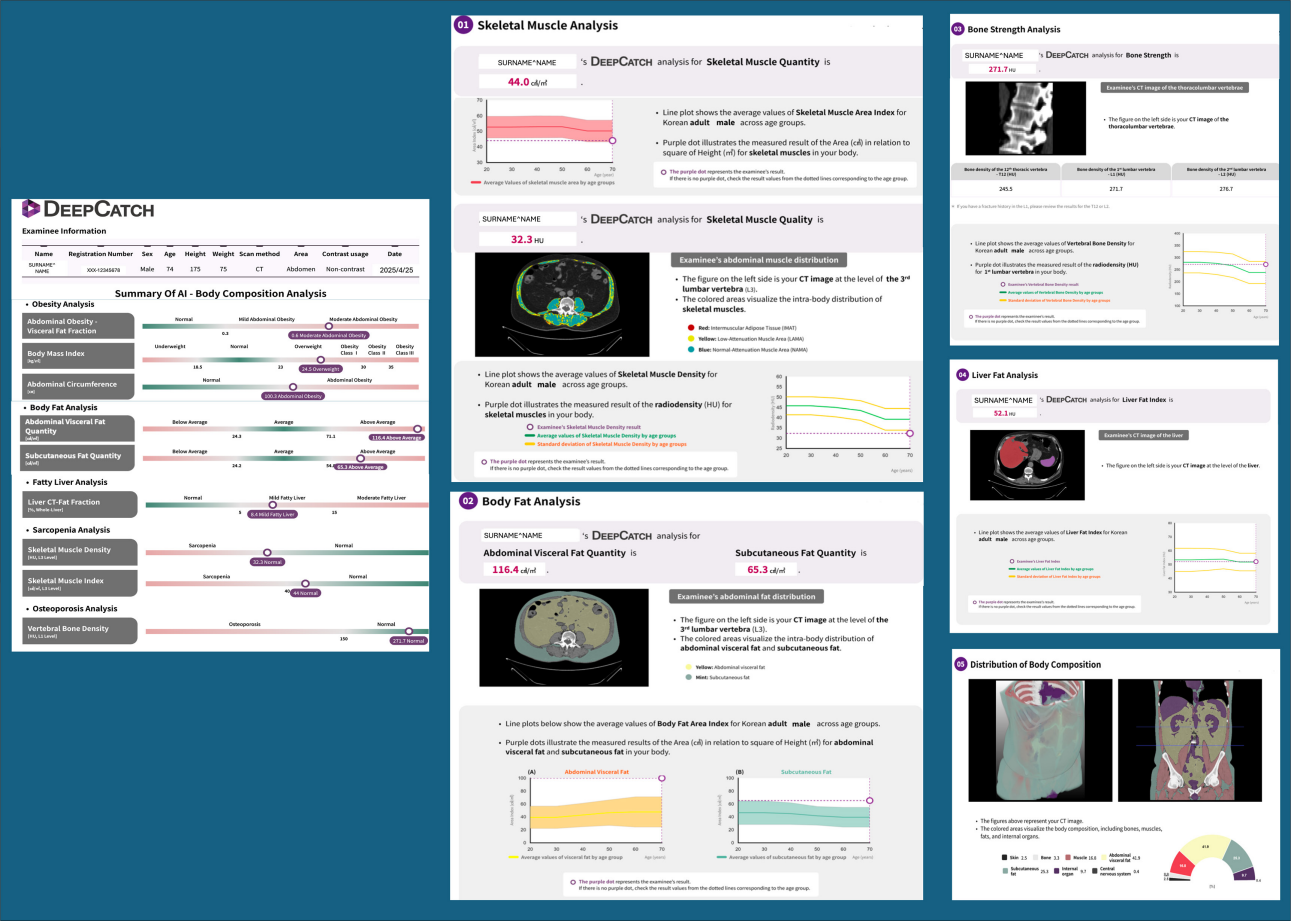
Example of an excerpt of body composition analysis (BCA) modified by a report generated by DeepCatch. The report presents a detailed summary of AI‐based body composition parameters, including assessments of obesity, body fat, liver fat, sarcopenia and osteoporosis status with corresponding quantitative values, range recognized as normal and classifications. Furthermore, there are subsections (1–5 circles upper left) with insights, graphs and quantitative data on the various sections. DeepCatch is a South Korean company specializing in advanced software for body composition analysis.

**FIGURE 5:**
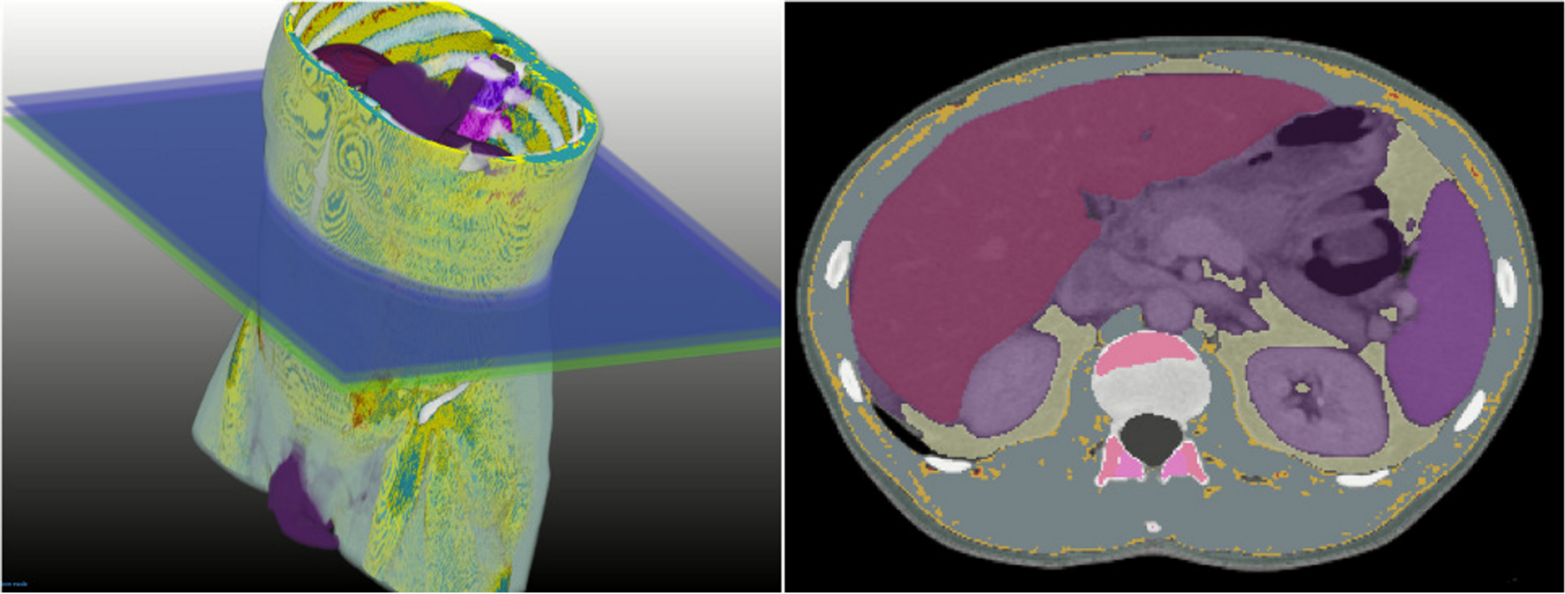
Volumetric model of body composition and segmentation of an axial abdominal section created using DeepCatch software (DeepCatch v1.2.0.0, MEDICALIP Co. Ltd., Seoul, South Korea). The image highlights the distinction between adipose, muscular, and bone tissues for a detailed quantitative assessment.

Therefore, the integration of AI and ML not only improves the analysis of medical imaging but also streamlines the diagnostic process as radiology practice becomes more demanding. This effectively reduces the workload for radiologists and promises a significant improvement in efficiency and accuracy in the field, including in the segmentation task helpful for different aspects including BCA [75]. An example of AI applied for BCA is provided in Figures 4 and 5.

### 6.1 AI: The Magic Bullet in BCA

BCA examines bodily tissues, while AI automates tasks and extracts insights, making them complementary in radiology; in other words, AI enhances BCA, while BC data supports AI development and algorithm context. This synergy enables the extraction, aggregation, and analysis of the multitude of data of body composition (not only VAT, SAT and lean body weight), overcoming the actual abovementioned limitations that relegate advanced BCA for research purposes only. The capability of AI systems to quickly and automatically extract a large amount of BC biomarkers and to build combined models would unlock the potential of BC metrics and the use of such data in a more accessible way in different branches of clinical practice to support clinicians with valuable information on the patient's body composition status to be integrated into the specific pathology. The growing literature highlights their synergy: BC path to establishing a standardized BC routine has gained renewed attention with AI's rise. However, a gap remains between speculative research and practical deployment of these tools.

The challenges in establishing a standardized BC routine range from technical issues (like the lack of consistent, objective parametrized tools and the limited availability of ad hoc datasets for algorithm development) to resistance in embracing change [76, 77]. Nonetheless, AI‐based solutions offer rapid, accurate and user‐friendly alternatives, such as the BodySegAI software, which significantly streamlines BCA workflows. In fact, the two‐dimensional U‐Net proposed for BodySegAI was trained and validated on 2989 CT scans with human segmentation as ground truth and finally also tested on 300 CT scans already analysed by an AI open‐source tool (AutoMATiCA); BodySegAI U‐Net resulted on average 148 times faster than manual performance with median DICE scores of 0.969 (skeletal muscle), 0.814 (IMAT), 0.986 (VAT) and 0.990 (SAT), also outperforming AutoMATiCA across all compartments, indicating superior segmentation performance [78]. The aforementioned limitations include the use of studies with a difference in methodology, small sample size and retrospective study design [77, 79]. Currently, despite the urgent need, a few studies provide a head‐to‐head comparison among the different AI BCA tools. In particular, an interesting study tested three different fully automated AI tools for abdominal CT‐based body composition analysis on a heterogeneous dataset of 8949 patients scanned on 83 different CT scanner types from six different vendors. All three tools resulted technically adequate in 97.7% of the examinations, highlighting their feasibility and robustness, potentially already applicable in real‐world conditions [80]. However, a comprehensive updated comparison among all the available AI BCA tools is not yet present in the scientific literature, with direct tests performed on the same imaging data. In the future, it is desirable that the tools developed provide comparable metrics (e.g., DICE score) and are tested on biobanks available online, so that rapid and effective feedback can be obtained on their functioning and comparison with other tools tested in the same way.

**FIGURE 6:**
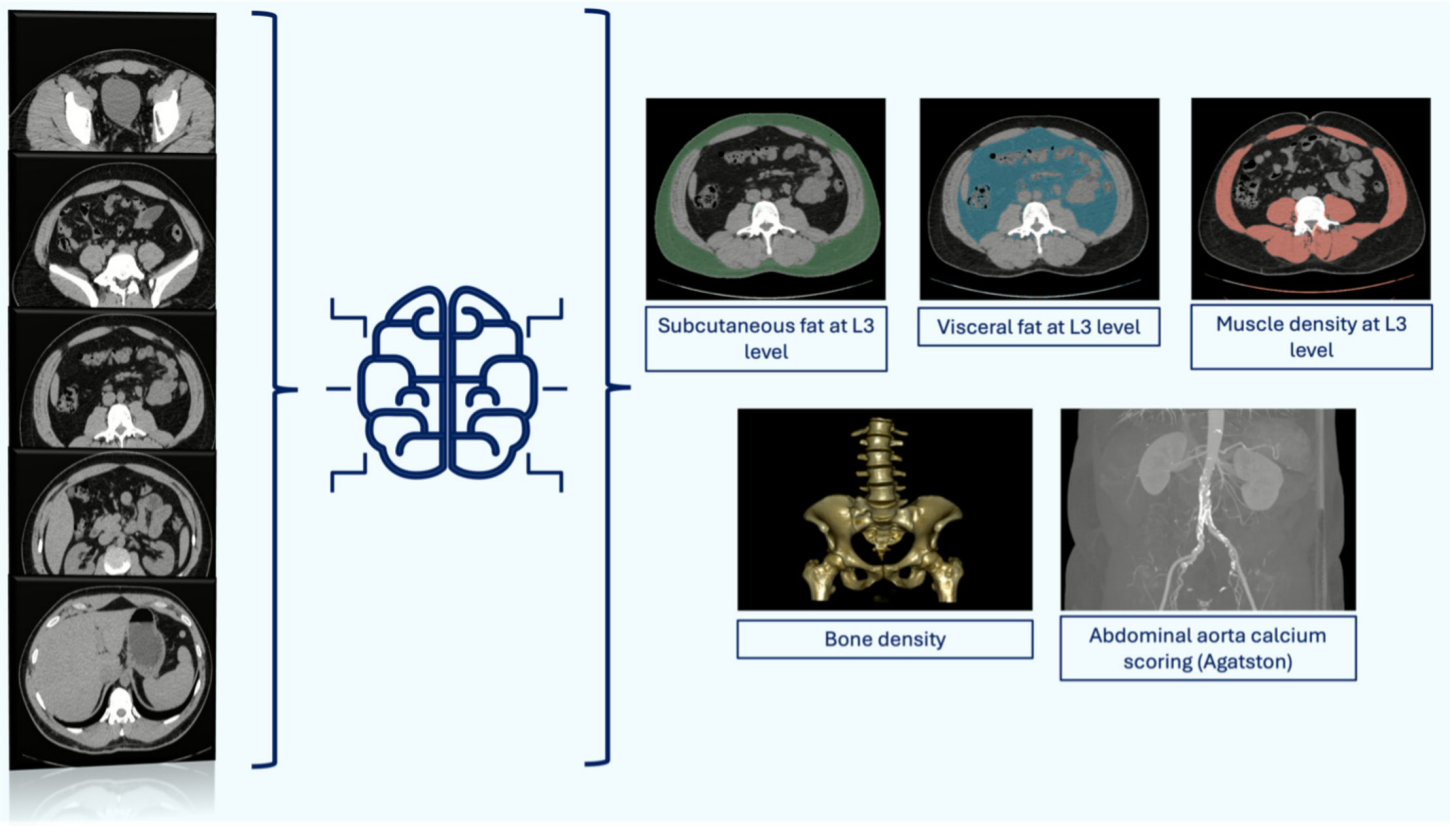
Example of body composition (BC) assessment with the artificial intelligence (AI) support. Thanks to AI is possible to extract different BC parameters with automatic segmentations and provide a complete BC assessment in a precise and quick manner from routinely acquired computed tomography.

Therefore, future research should be directed towards the standardization of the assessment protocol, cross‐cultural generalizability and validation of the AI model in different populations and interdisciplinary cooperation in the creation of large and high‐quality datasets [76]. Moreover, ongoing development in the fields of explainable AI and transparent algorithmic systems is paramount to foster trust and ensure more integration of AI‐based BCA into clinical practice [81]. Another important aspect for the clinical applicability is the regulatory field. Nowadays, only a few AI tools for body composition analysis have received the FDA clearance (i.e., DeepCatch CT‐based and Prenuvo's MRI platform) [82], whereas in Europe, such tools should fall under the new AI Act and MDR framework as high‐risk medical devices, underlining both their high clinical potential impact and the urgent need for fully implemented, harmonized regulations, not yet completely established [83]. Overcoming clinical AI adoption barriers is key to fully realizing AI's potential in BCA (Figure 6). Taking into consideration all these aspects, we propose a possible checklist as a framework for clinical integration and a pragmatic tool to appraise readiness before adopting a specific algorithm in practice (Table 1).

**TABLE 1:** Proposed checklist for AI body composition Analysis tools assessment.

Domain	Checklist item	What to verify/evidence to cite
Use case	□ Intended use and target population defined	Opportunistic CT vs. BC dedicated protocol; inpatient/outpatient; oncology/general practice
	□ Task alignment and interpretable outputs	Segmentation/measurement vs. prediction; clinically meaningful outputs (i.e., SM area/SMI, VAT)
Evidence and generalisability	□ Human reference standard	Ground truth by expert readers; clear manual protocol/landmarks (e.g., L3)
	□ External validation	Tested on data from other sites/scanners; type of population tested
	□ Fairness across subgroups	Reported performance by sex, age, BMI, ethnicity/disease where relevant
Benchmarking	□ Head‐to‐head vs. validated tool	Direct comparison against a clinically used, FDA‐cleared or CE‐marked tool for the same task/population, using the same reference standard, metrics, and dataset. Report superiority/non‐inferiority with pre‐specified margins; justification needed if not available
Performance and robustness	□ Accuracy	Overlap (Dice/HD), agreement (ICC/Bland–Altman); if predictive, AUC/HR vs. outcomes
	□ Robustness and failure modes	Sensitivity to artefacts/contrast phases/incomplete FOV; documented failure cases and fall‐back plan
Reproducibility and versioning	□ Repeatability	Test–retest or scan–rescan data; intra/inter‐study consistency
	□ Version control	Clear software versioning, change logs, and model update policy
Sarcopenia‐specific suitability	□ Definitions and thresholds	Align with recognized criteria (e.g., EWGSOP/AWGS) or provide institution‐specific cut‐offs with rationale
	□ Outcome linkage	Associations with sarcopenia‐relevant outcomes (frailty, complications, LOS, survival) in comparable cohorts
Integration and workflow	□ Interoperability	PACS/RIS integration, DICOM Seg/SR export; turnaround time compatible with clinical workflow
	□ Human oversight and QA	Visual mask overlays; who reviews/approves; escalation pathway for uncertain cases
Governance and regulation	□ Regulatory status	Intended use matches FDA clearance/CE mark (or local equivalent); privacy and cybersecurity documented
	□ Local validation plan	Acceptance thresholds and sample size before go‐live
Monitoring and QA	□ Post‐deployment monitoring	Drift checks, periodic audits, incident logging, re‐validation after updates
Economics and practicality	□ Net benefit and resources	Time/cost savings, throughput, GPU/IT needs, support availability
Reporting and transparency	□ Reporting standards	Adheres to TRIPOD‐AI/CLAIM/CONSORT‐AI as applicable; documentation accessible to end‐users

Abbreviations: AI, artificial intelligence; AUC, area under the (ROC) curve; BC, body composition; CE, Conformité Européenne (CE mark); CT, computed tomography; Dice (DSC), dice similarity coefficient; DICOM, Digital Imaging and Communications in Medicine; EWGSOP/AWGS, European Working Group on Sarcopenia in Older People/Asian Working Group for Sarcopenia; FDA, US Food and Drug Administration; FOV, field of view; GPU/IT, graphics processing unit/information technology; HD, Hausdorff distance; HR, hazard ratio; ICC, intraclass correlation coefficient; LOS, length of stay; PACS, picture archiving and communication system; QA, quality assurance; RIS, radiology information system; Seg/SR, segmentation object/structured report (DICOM); SM, skeletal muscle; SMI, skeletal muscle index; VAT, visceral adipose tissue.

### 6.2 BC and AI‐Based Segmentation

In BC, the most important AI application is automating tissue segmentation, which is tedious and prone to human error due to the large amount of data. AI can speed up the process and assist radiologists in their supervisory role [84]. For example, a recent systematic review by Mai et al. [76] proved the feasibility of several DL algorithms in clinical practice to provide fully automated volumetric BCA from 3D CT scans, improving the accuracy of human supervisors.

Another work by Faron et al. [85] demonstrated the feasibility of DL‐based BCA to predict relevant endpoints in melanoma patients; also, Hsu et al. [48] showed in their study the effectiveness of another DL algorithm in classifying patients diagnosed with pancreatic cancer according to their mortality risk, starting from imaging findings. The AI model measured fat and muscle mass in pancreatic cancer patients, linking sarcopenia to mortality with a mean survival of 15 ± 12 versus 22 ± 12 months (*p* < 0.05). Also, it is worth noticing that several studies have compared the speed of segmentation between human operators and automatic algorithms: In a recent study from Gomez‐Perez et al. [86], the automatic segmentation algorithm showed excellent concordance for L3 BC parameters with a semi‐automated method led by a human operator (the so‐called ‘ground truth’ that serves as a reference base). Other studies reported similar conclusions, showing that increasing training and data access improve algorithm accuracy and speed [87, 88].

The boost provided by AI to the BCA is also noticeable, not only for baseline diagnosis but also for follow‐up. An interesting study conducted by Borelli et al. highlights how sarcopenic status at baseline and follow‐up in patients with urothelial tumours can be easily assessed with an AI‐powered tool on CT images and potentially enable the monitoring of sarcopenic status that can be managed with customized nutritional therapies to improve the outcome [89]. A deeply representative example of the impact of AI on muscle assessment is then provided by the study proposed by Pickhardt et al. through the analysis of 9223 asymptomatic adults who underwent abdominal CT. The study aimed at comparing the utility of DL CT‐based muscle quantification at the L1 and L3 level for the prediction of future hip fractures and death with an average of 8.8 years of follow‐up. Results showed the comparable metrics of muscle attenuation measurement (myosteatosis) on both L1 and L3 compared with established clinical risk scores, expanding the possibility to assess this parameter in an opportunistic manner not only on abdominal CT but also on CT thoracic examination [90].

As a matter of fact, automatic segmentation has been shown to be significantly faster than manual segmentation. Beetz et al. [91] showed that AI‐based segmentation was 82 times faster than semi‐automatic methods. So it is clear how these advancements are pivotal for streamlining the radiologist's workflow, enhancing both efficiency and accuracy.

AI also extends to MRI, where DL protocols are developed for tasks like liver segmentation. Yan et al. [92] proposed a framework to extract liver segmentation for clinical use and quantitative fat analysis. Recently, Schneider et al. [93] demonstrated that DL approaches for adipose tissue quantification derived from MRI data are attainable even for patients with obesity, with a resulting accuracy that is equal to or better than methods led by human operators. In recent years, the application of these technologies in clinical practice has become so widespread that the Chinese Medical Association has already reached a consensus on the clinical practice guidelines to apply for BC assessment regarding MR imaging. Very impressive results were recently published by Jung et al., who developed a DL method to assess BC from whole‐body MRI in a population of more than 50 000 patients from two different cohorts, which reached a Dice coefficient ranging between 0.86 and 0.88 [94].

It is evident that research on the use of AI is heavily focused on segmentation, as it is the main task in BCA and the most operator‐dependent one [95]. It is noteworthy to mention also that Kelly et al. [96] appraised the necessity to further research all the possible applications of AI because the segmentation task is the most prominent, but not the only one.

### 6.3 Other Tasks

Nonetheless, besides segmentation, these instruments are employed for different operations: ML algorithms have been shown to outperform humans in choosing representative pictures for examination from an axial array (such as mid‐L3 vertebral body) when it comes to BCA [97]. Innovative projects include Graybel et al. and their AI‐based smartphones to estimate BC from standard parameters [98]. In particular, AI for sarcopenia and BC can be implemented for decision‐support models that combine BC metrics with clinical data. Examples include algorithms developed to predict sarcopenia, lean and fat mass from demographic and anthropometric data [99, 100], ultrasound AI‐based elastography models that use muscle stiffness and ultrasound grayscale to infer muscle quality [101] and radiomics muscle data and clinical biomarkers integrated with demographics and labs to build a predictive nomogram. In oncology and perioperative care, several nomograms incorporating sarcopenia and adiposity measures with other routine variables improve risk stratification for postoperative complications, survival or treatment tolerance [102]. Furthermore, a very promising example in the field of sarcopenia treatment is provided by Reza et al. [103], who proposed an attention‐aware multi‐task learning framework that integrates multi‐omics layers (SNVs, mRNA expression and DNA methylation) to prioritize therapeutic targets and repurpose sarcopenia treatments, selecting canagliflozin as a possible candidate, shifting AI's role from phenotyping and descriptive tool to treatment hypothesis generation. Globally, these approaches associated with the segmentation task tools show how AI can operationalize body composition analysis in a new and implemented manner in the field of precision medicine.

As AI advances, it will provide more detailed and precise assessments. It is not hard to think that AI will play a crucial role in standardizing BCA, ensuring consistent and reproducible results across various patients and imaging sessions. This standardization process may be vital for longitudinal studies and for comparing results across different healthcare facilities, paving a new way into this research field.

## 7 AI Tools Application in Current Healthcare: Real‐Life Problems from the Lab to the Ward

Today, AI is ubiquitous, and recently, it has also been integrated into medicine to expedite processes and/or enhance patient care [104]. However, if AI's roots extend so far back as the abovementioned, why does it seem to be a recent sensation? The answer lies in the context in which AI has flourished. AI is a powerful tool, but it requires data and a substantial workflow orchestration to function effectively; otherwise, it remains merely an excellent idea. Therefore, a significant challenge must be addressed: transforming the context in which AI must be embedded to realize its full potential, as happened in the past for World Wide Web and Big Data.

By resolving the initial challenges in AI software development (already addressed once a product becomes market‐ready), the focus should shift to tackling issues related to integrating AI into hospital systems, particularly those involving AI‐based BC tools.

To provide an objective point of view on the topic, some surveys have been conducted to identify the possible hurdles present in clinical routine, highlighting how AI acceptance is influenced by generational factors and underscoring the paramount importance of education to effectively engage with AI and integrate this technology into daily work. Furthermore, a vast part of professionals found AI reliable; however, a neat impact on workload reduction has not been perceived yet, as only one‐third of the interviewed referred to the use of these tools. Huisman et al. performed a survey involving 1041 certified board radiologists and radiology residents from 54 predominantly European countries. Only 48% of the participants showed an open attitude towards AI; this was especially true for those with non‐basic AI knowledge. Conversely, 30% were apprehensive about AI replacing their jobs, while 23% questioned their career choice [105]. The latter part of the same study revealed that 62% identified ethical and legal issues as barriers to AI adoption, underlining the necessity of including AI education in radiology curricula [106]. However, a different survey painted a contrasting picture: medical students generally showed little concern regarding AI taking over the role of radiologists and recognized potential applications [107]. Surprisingly enough, only 17.8% of the European Society of Radiology (ESR) survey population (*n* = 690) faced technical integration issues with AI tools while 75.7% found diagnostic algorithms reliable, 34.6% mentioned algorithm use and 17.3% disclosed it to patients. Workload reduction was noted by 22.7%, with 69.8% seeing no effect. For workflow prioritization, 23.4% found algorithms very helpful, 62.2% moderately helpful and 13.3% intended to acquire AI tools [108]. Moreover, it was also indicated that 73.3% (*n* = 198/270) of radiologists felt they had insufficient AI training, but 79.3% (*n* = 214/270) believed AI would positively impact their future practice, anticipating reductions in medical errors (81%) and interpretation time (74.4%) [109].

Key problems identified are skepticism about AI's value, fear of job replacement, educational gaps, ethical and legal concerns and integration challenges (Table 2).

**TABLE 2:** Summary of fundamental problems and possible solutions for AI integration in healthcare, focusing on hospital systems and radiology.

Real life problems	Critical issues	Possible solutions
User acceptance	Scepticism about AI's value [110]. Fear of job replacement [105]. Mistrust [110, 111].	Education and training in AI for radiologists and medical students [112, 113]. Involvement of ‘local champions’ to promote AI adoption [114].
Educational gap	Lack of AI knowledge among radiologists [110]. Limited access to training and mentorship opportunities [111].	Incorporation of AI courses in medical and radiology curricula [106]. Continuous training on AI efficacy, cost‐effectiveness, patient safety, and accuracy.
Ethical and legal issues	Ethical and legal concerns as barriers to AI adoption [106].	Inclusion of ethical and legal aspects of AI in educational programs [106].
Technical integration	Difficulties integrating AI with existing IT systems [105, 106, 108]. Technological incompatibilities [105].	Development of robust IT infrastructure capable of handling large data volumes and supporting AI integration [107].
Organizational readiness	Lack of organizational preparedness for AI adoption [115].	Creation of inter‐organizational networks to share knowledge and experiences [114]. Use of the NASSS framework to evaluate AI application readiness.
Financial barriers	High costs of implementation and maintenance [116]. Lack of reimbursement models (124).	Establishment of sustainable funding models [116]. Encouragement of reimbursement for AI use, similar to the adoption of telemedicine during COVID‐19.
Practical effectiveness	No significant impact on workload reduction [108].	Smooth integration of AI into clinical processes to improve operational efficiency and diagnostic accuracy [117].

Abbreviations: AI, artificial intelligence; IT, information technology; NASSS, non‐adoption, abandonment, scale‐up, spread and sustainability.

### 7.1 The Human Capital: Between Hesitation and Confidence, Between Underestimation and Education

Since civilization began, new inventions often faced initial scepticism: AI, as a black‐box technology, eludes logical explanation, leading to hesitations and suspicion regarding its implementation. The primary reason for radiologists' mistrust is a lack of AI knowledge [110], compounded by limited access to mentorship and training [111].

To resolve this hesitation, a fundamental know‐how of ML and AI in general is essential [112]. Radiology residents should acquire basic AI knowledge to troubleshoot AI malfunctioning, similar to how they manage image artefacts [113]. Then, once education meets these needs, educators must stay prepared for automation complacency and anchoring biases that occur when clinicians rely too heavily on AI without rigorous evaluation. Continuous AI training should cover efficacy, cost‐effectiveness, patient safety and technical accuracy. A key solution involves ‘local champions’ within institutions, such as motivated residents or experienced radiologists, who advocate for AI and facilitate its implementation [114]. Until medicine and radiology curricula include AI courses [106], local champions will lead AI deployment and drive its implementation within departments.

### 7.2 The Infrastructure: Not Only Health

One of the major real‐life obstacles is integrating AI into existing IT workflows. This process should not only be seamless but also reduce the workload. When integrating an AI algorithm, prioritizing IT infrastructure and the hospital environment is crucial; key factors include the ability to process large data volumes and ensure support, which entails significant costs. Technological incompatibilities have prevented AI from analysing up to 22% of research in certain clinical scenarios, highlighting the need for robust IT infrastructure to manage data and integrate AI effectively. Huisman et al. add that smooth workflow integration is crucial for the effective adoption of AI but is frequently disregarded [105, 106]; in a word, organizational readiness. This is a definition discussed thoroughly by Alami et al. and indicates an essential organizational preparation, which includes institutional resources, a supportive organizational atmosphere and motivational preparedness, that must be achieved before such a demanding change takes place [115].

Cost is another barrier preventing AI from being widely used in healthcare. It has been emphasized how ongoing funding is required to support software and hardware as well as AI system maintenance, even if well‐defined repayment plans are not common practice [116]. Strategic solutions are needed for these problems, but achieving more funding requires validation, which is challenging due to existing issues. Successful AI integration in healthcare needs sustainable funding models, a strong IT infrastructure and organizational readiness through collaborative efforts and strategic leadership. AI systems must be smoothly incorporated into clinical processes to detect and report examination quality issues, initiate image analysis promptly and produce preliminary findings for radiologists to review [117].

Integrating AI into clinical practice involves seemingly prohibitive licensing costs, at least initially. However, from a prospective viewpoint, focusing on early diagnosis, where AI‐based BCA can provide maximum support to human physicians, it is essential to consider the economic savings. For instance, a study demonstrated that AI‐assisted CT‐based opportunistic screening for cardiovascular events, osteoporosis and sarcopenia resulted in cost savings by preventing symptomatic events and reducing healthcare costs associated with these conditions. Thus, the initial high costs of AI can be justified by the substantial economic benefits from reduced treatment costs and improved patient outcomes over time [118]. By addressing these issues, AI's potential to improve overall healthcare outcomes, operational efficiency and diagnostic precision will be realized.

## 8 Conclusions

In conclusion, to effectively narrow the gap between research studies on BC and clinical practice, AI seems to represent the ideal tool able to overcome the actual limits. To become reality, the union between BC and AI will need concerted efforts to establish international standards, rigorous validate AI technologies in real‐world settings, improve educational initiatives and foster collaborative networks among stakeholders.

By addressing these challenges systematically, healthcare systems can leverage AI to improve BC in accuracy, operational efficiency and patient outcomes, thereby ushering in a new era of personalized and effective healthcare delivery.

## Funding

The authors received no specific funding for this work.

## Ethics Statements

The manuscript does not contain clinical studies or patient data.

## Conflicts of Interest

The authors declare no conflicts of interest.

## Supporting information


**Data S1:** supplementary information.

**Figure S1:** An example of the magnetic resonance imaging sequences used in body composition; a multi‐echo sequence (Iterative Decomposition of water and fat with Echo Asymmetry and Least squares estimation‐ IDEAL‐IQ‐ GE Healthcare) that allows an advanced chemical‐shift encoded fat quantification method corrected for confounding factors such as T2* effect. (a) water only reconstruction (b) fat only reconstruction.

**Figure S:** Proton Density Fat Fraction (PDFF) image reconstruction allowing fat fraction estimation expressed in percentage; red region of interest (ROI) is placed in the liver parenchyma.

**Table S1:** Summary of the main differences between the imaging techniques: DEXA, CT and MRI.
