# Supplementary material for: From Pixels to Prediction: Reviewing the Role of Artificial Intelligence in Body Composition Analysis
Source: J Cachexia Sarcopenia Muscle. 2026 May 18;17(3):e70218. doi: 10.1002/jcsm.70218 (PMC13181601; doi:10.1002/jcsm.70218)
Supplement: Supplementary file 1 — Data S1: supplementary information. [file JCSM-17-e70218-s003.docx]

**Supplemental Material**

**Caption Figure Supplemental Material**

**Fig. S1** An example of the magnetic resonance imaging sequences used in body composition; a multi-echo sequence (Iterative Decomposition of water and fat with Echo Asymmetry and Least squares estimation- IDEAL-IQ- GE Healthcare) that allows an advanced chemical-shift encoded fat quantification method corrected for confounding factors such as T2* effect. a) water only reconstruction b) fat only reconstruction

**Fig. S2** Proton Density Fat Fraction (PDFF) image reconstruction allowing fat fraction estimation expressed in percentage; red region of interest (ROI) is placed in the liver parenchyma
