# Supplementary figures and images for: From Pixels to Prediction: Reviewing the Role of Artificial Intelligence in Body Composition Analysis
Source: J Cachexia Sarcopenia Muscle. 2026 May 18;17(3):e70218. doi: 10.1002/jcsm.70218 (PMC13181601; doi:10.1002/jcsm.70218)

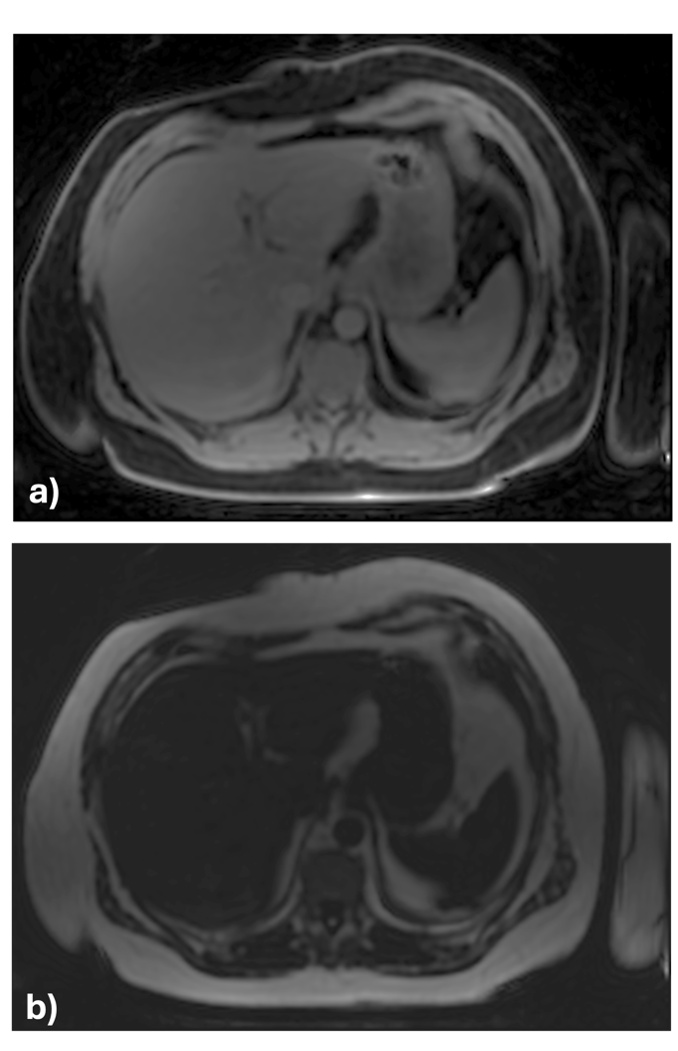

Supplement: Supplementary file 2 — Figure S1: An example of the magnetic resonance imaging sequences used in body composition; a multi‐echo sequence (Iterative Decomposition of water and fat with Echo Asymmetry and Least squares estimation‐ IDEAL‐IQ‐ GE Healthcare) that allows an advanced chemical‐shift encoded fat quantification method corrected for confounding factors such as T2* effect. (a) water only reconstruction (b) fat only reconstruction. [file JCSM-17-e70218-s004.png]

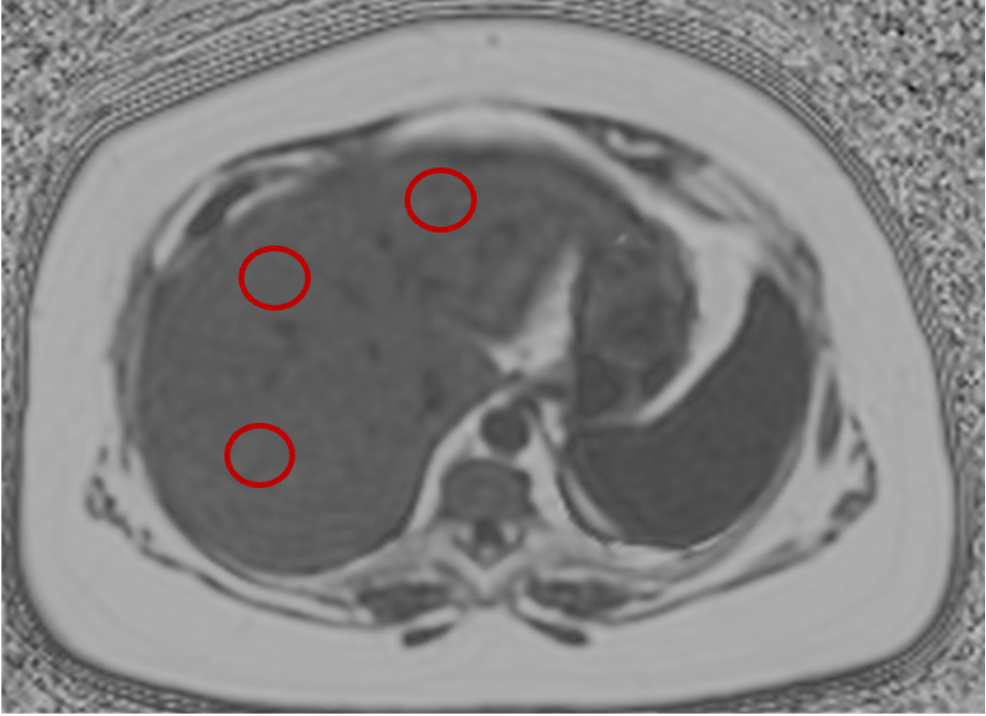

Supplement: Supplementary file 3 — Figure S: Proton Density Fat Fraction (PDFF) image reconstruction allowing fat fraction estimation expressed in percentage; red region of interest (ROI) is placed in the liver parenchyma. [file JCSM-17-e70218-s001.png]
