# Supplementary material for: From Pixels to Prediction: Reviewing the Role of Artificial Intelligence in Body Composition Analysis
Source: J Cachexia Sarcopenia Muscle. 2026 May 18;17(3):e70218. doi: 10.1002/jcsm.70218 (PMC13181601; doi:10.1002/jcsm.70218)
Supplement: Supplementary file 4 — Table S1: Summary of the main differences between the imaging techniques: DEXA, CT and MRI. [file JCSM-17-e70218-s002.docx]

**Table S1.** Summary of the main differences between the imaging techniques: DEXA, CT and MRI

| **Imaging technique** | **Main aims** | **Advantages** | **Disadvantages** |
| --- | --- | --- | --- |
| **DEXA** | - Assess body composition by evaluating fat, lean mass, and bone mineral density (BMD) (36). | - Can assess lean body mass and body fat of a single body region or the whole body, having the two constant attenuation coefficients (R-value) (18). - Lower radiation exposure than CT. | - Influenced by body thickness and water status, since it cannot distinguish water from lean mass (38). - Non-portable and variability in measurements between different machines and software (40). |
| **CT** | - Quantification of VAT, SAT, BMD, muscle mass, and aortic calcifications (39). | - Provides high-quality images and tissue-specific thresholds (38). - Assess intramuscular fat and, to a lesser extent, liver fat (18). - Compared to MRI: less expensive, shorter scanning time, better at detecting calcification. | - Involves radiation exposure. - High cost of examination (38). - Requires adequate preparation for image segmentation (18). - Limited ability to assess low-grade liver steatosis (<5%) (18). |
| **MRI** | - Quantification of adipose tissue and lean mass, or adipose infiltration of an organ or tissue (18). | - No ionizing radiation. - Allows detailed analysis of muscle fat and visceral fat infiltration (43). - Dixon sequences allow the quantification of muscle fat and visceral fat infiltration (18, 43) - MRI-Spectroscopy provides chemical and molecular info about the tissue (42, 43) | - High cost and longer examination times (42). - Limited accessibility for patients with non-compatible metal devices (42). - Less availability. |
| *DEXA: Dual Energy X-Ray Absorptiometry; CT: Computed Tomography; MRI: Magnetic Resonance Imaging; BMD: Bone Mineral Density; VAT: Visceral Adipose Tissue; SAT: Subcutaneous Adipose Tissue.* | | | |
